# Oncolytic virotherapy: new weapon for breast cancer treatment

**DOI:** 10.3332/ecancer.2020.1149

**Published:** 2020-12-03

**Authors:** Veronica Martini, Francesca D’Avanzo, Paola Maria Maggiora, Feba Maria Varughese, Antonio Sica, Alessandra Gennari

**Affiliations:** 1 Division of Oncology, Department of Translational Medicine, University of Eastern Piedmont, Novara 13100, Italy; 2 Center for Translational Research on Autoimmune & Allergic Diseases – CAAD, Novara 28100, Italy; 3 Department of Pharmaceutical Sciences, University of Eastern Piedmont, A Avogadro 28100, Italy; 4 Department of Inflammation and Immunology, Humanitas Clinical and Research Center–IRCCS, Rozzano (MI) 20089, Italy; a https://orcid.org/0000-0002-0887-4082; b https://orcid.org/0000-0002-8342-7442; c https://orcid.org/0000-0002-0928-2281

**Keywords:** OVs, immunotherapy, BC

## Abstract

The recent introduction of viruses as a weapon against cancer can be regarded as one of the most intriguing approaches in the context of precision medicine. The role of immune checkpoint inhibitors has been extensively studied in early and advanced cancer stages, with extraordinary results. Although there is a good tolerability profile, especially when compared with conventional chemotherapy, severe immune-related adverse events have emerged as a potential limitation. Moreover, there are still treatment-resistant cases and thus further treatment options need to be implemented. Several *in vitro* and *in vivo* studies have been conducted and are ongoing to develop oncolytic viruses (OVs) as a tool to modulate the immune system response. OVs are attenuated viruses that can kill cancer cells after having infected them, producing microenvironment remodelling and antitumour immune response. The potential of oncolytic virotherapy is to contrast the absence of T cell infiltrates, converting ‘cold’ tumours into ‘hot’ ones, thus improving the performance of the immune system. Breast cancer, the second most common cause of cancer-related deaths among women, is considered a ‘cold’ tumour. In this context, oncolytic virotherapy might well be considered as a promising strategy. This review summarises the current status, clinical applications and future development of OVs, focusing on breast cancer treatment.

## Background

The immune system plays an important role in controlling and eradicating cancer. However, malignant cells can develop multiple mechanisms of immune suppression [1]. In recent years, a number of immunomodulating agents have been developed as anticancer therapies and introduced into the clinical practice. Among these, immune checkpoint inhibitors (ICIs) have demonstrated an impressive level of activity as antitumour treatment in different tumour types [2], such as advanced melanoma, non-small cell lung cancer and breast cancers [2, 3]. The major mechanism of action involves the removal of inhibitory pathways that block effective anti-tumour T cell responses; as a consequence, the immune system is re-educated to fight against cancer cells. Yet, the activity of this class of agents is heterogeneous within the same tumour type, and innovative and complementary strategies need to be developed. In particular, tumour microenvironment (TME) might exert an important role in conditioning the immune response. In fact, it has been shown that the so-called hot tumours, characterised by the presence of Tumour-Infiltrating Lymphocytes (TILs), expression of programmed death-ligand 1 (PD-L1) on tumour-associated immune cells and possible genomic instability have the higher chance of response [4]. Conversely, ‘cold’ tumours scarcely expressing PD-L1, characterised by low mutational burden (low expression of neoantigens) and low expression of antigen presentation markers, such as major histocompatibility complex class I (MHC I) [4] are considered less responsive. In this scenario, the treatment of immune ‘cold’ tumours, such as tumours of the breast, prostate, ovary, pancreas and others, with this class of agents may be characterised by suboptimal responses and resistance. In this perspective, different approaches have been identified to overcome the absence of T cell infiltrates, thus converting ‘cold’ tumours into ‘hot’ ones. Among these, chemotherapy has been evaluated as a possible strategy to reshape tumour milieu and get immune infiltrate compatible with an expected activity of the ICI treatment [5, 6]. Many studies are also exploring the ability of viruses to kill tumour cells by initiating systemic immune responses through different mechanisms such as induction of Immunogenic Cell Death (ICD), release of danger signals (damage-associated molecular pattern (DAMPs)) and of tumour antigens from virus-infected cells [7].

Oncolytic Viruses (OVs) are native or genetically modified viruses that selectively infect and replicate within tumour cells, eventually leading to tumour cell lysis. Alongside this direct and local antitumour activity, OVs can also induce a potent, systemic and potentially durable antitumour immunity. The dying tumour cells release a lot of factors that engage antitumour immunity, inducing therapeutic responses also at distant tumour sites [7].

Several OVs have been successfully tested in different preclinical models [8]. In 2015, the Food and Drug Administration (FDA) and European Medicines Agency (EMA) approved the use of talimogene laherparepvec (T-VEC, Imlygic®) as oncolytic viral therapy for advanced melanoma patients [9]. In glioma, several OVs have clearly demonstrated both safety and a promising efficacy in the phase I clinical trials [7]. Various OVs, such as HF10 (Canerpaturev—C-REV) and CVA21 (CAVATAK), are now actively being developed in phase II as monotherapies, or in combination with ICI against melanoma [10]. The clinical trials of OVs against pancreatic cancer have not yet demonstrated efficacy as either monotherapy or as part of combination therapy [7].

This review provides an overview on the development of oncolytic virus immunotherapy. In the first part, we briefly describe the mechanisms of immunosurveillance that are the basis on which the OVs are built and developed. In the second part, we discuss preclinical and clinical data that show a role for OVs in breast cancer therapy.

## Cancer cells and host immune responses: mechanism of immunosurveillance

**Figure 1.**
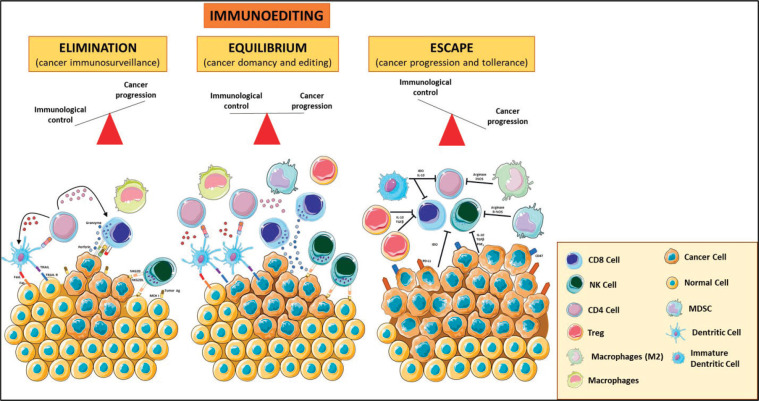
Immunoediting. The three phases of the cancer immunoediting process: elimination, equilibrium and escape. In tumour elimination (left), which often occurs in early tumour development, highly antigenic tumour clones are recognised and eliminated by both innate and adaptive immune systems. In the equilibrium phase (middle), the tumour and the adaptive immune system coexist. Tumour escape (right) occurs when there is poor antigenic expression, immunosuppressive cytokines, MDSCs, and expression of negative regulatory receptors. Various forms of immunotherapy aim to shift the balance from escape and equilibrium to elimination. Treg: T regulatory; MDSCs: Myeloid Derived Suppressor Cells; MCH I: Major Histocompatibility Complex Class I. The figure was modified from Servier Medical Art, http://smart.servier.com/.

TME is composed of many cell types, including the original cancer cell with genetic alterations and other cells, such as fibroblasts, endothelial cells, and eventually a variety of immune cells that can mount a response against tumour cells. Those tumour cells that escape from immune system attack can establish an immunosuppressive microenvironment. This highly dynamic immunoediting process is defined by three phases: (1) the elimination phase (i.e., immunosurveillance), where the malignant cells are detected and cleared by immune cells; (2) the equilibrium/editing phase, where there is a continuous T-cell-mediated eradication of malignant cells via effector responses including CD8+ T-cells, γδ T-cell subsets and Natural Killer (NK) cells, as well as macromolecules including interferon g (IFNγ), perforin, and TNF-related apoptosis-inducing ligands and (3) the escape phase, characterised by uncontrolled cell growth and proliferation that can lead to metastatic spread [11]. TILs, tumour-associated macrophages (TAMs) and myeloid-derived suppressor cells (MDSCs) play an important role in the promotion and maintenance of tumour immune escape (Figure 1).

TILs were defined as lymphocytes that moved from the blood into the tumour bed and, directly opposing and/or surrounding tumour cells, can recognise and kill them. They include T, B and NK cells [12]. The interactions between TILs and cancer cells play a critical role in disease progression. Therefore, the role of TILs has been investigated in the TME. CD8+ and CD4+ T lymphocytes can be activated directly or indirectly by cancer cells: in the first case, tumour cells can present neoantigens by means of MHC-I and MHC-II molecules; alternatively, T cell activation can occur indirectly through dendritic cells (DC) or macrophages (M), killing, processing and presenting neoantigens to T lymphocytes [12]. TILs exert a good prognostic effect in patients treated with ICI (i.e., anti-PD-1/PD-L1 pathway) not only because they activate the antitumour immune response but also because they release IFNg, favouring PD-L1 expression in tumour cells and providing a positive feedback [13]. Activated cytotoxic CD8 T cells and NK cells have been associated with a good prognosis; in contrast, the presence of Treg has a negative effect on several cancers, due to the enhancement of tumour aggressiveness and suppression of antitumour immune responses [14]. In normal conditions, Treg cells are involved in maintaining immune homeostasis by protecting hosts against the development of allergic and autoimmune disorders [15]. In this regard, it has been demonstrated that enhanced presence of Treg cells in biopsy samples of the breast tumours is associated with an adverse relapse-free and overall survival [16]. Breast cancer is not considered to be immunogenic, due to several factors, including a low tumour mutational load [17]. Indeed, specific TIL subtypes have been associated with different outcomes in Triple-Negative Breast Cancer (TNBC) patients [17]: in particular, high levels of PD-1+ TILs or FOXP3+ TILs have been shown to correlate with a poor prognosis, whereas high levels of CD8+ TILs seem to be associated with an improved prognosis [18].

The reduction of effector functions of TILs, the promotion of T-reg and the secretion of inhibitory cytokines are the consequence of TAMs activity, thus promoting tumour growth and progression [19]. TAMs derive from peripheral blood monocytes recruited into the microenvironment and in response to several stimuli, such as inflammation, undergo M1 (classical) or M2 (alternative) activation. It is well known that some cytotoxic agents are able to favour the antitumour activities of TAMs, at least in leukaemia and/or immunogenic (transplant) tumour models [20]. In this regard, chemotherapy can induce ICD which is triggered by innate immune cells or infiltrating immune cells, including TAM and tumour-associated dendritic cells (TADCs). ICD activates TADC and TAM sustaining enhanced antigen presentation and supporting specific anti-tumour immunity [21]. In this scenario, phagocytosis of cancer cells by TAMs provides additional antitumour activity, which, however, is impaired by the ‘don’t eat’ me signal CD47, expressed by many cancer cells [20]. CD47 is a transmembrane glycoprotein with an inhibitory effect on phagocytosis, called ‘don’t eat me’ signal. This is mediated by CD47 binding to signal-regulatory protein (SIRPα), which is expressed on TAMs [22]. CD47 is overexpressed in many types of human cancer cells and protects them from being recognised and cleared by innate immune surveillance. Since high CD47mRNA expression is associated with poor survival of cancer patients [23], antibodies anti-CD47 were studied in various preclinical models and are currently being tested in ongoing clinical trials [24]. Our unpublished preliminary data, obtained by Immunohistochemistry (IHC), showed a major expression of both CD47 and SIRP1a in TNBC with respect to Luminal BC. Moreover, it was demonstrated that treatment with anti-human CD47 of cancer stem cells isolated from the Triple-Negative MDA-MB-231 cell line decreases proliferation and up-regulates tumour suppressor genes [25]. In this scenario, several TAM-centred strategies have been proposed, such as the suppression of TAM recruitment, the depletion of their number, the promotion of M1-polarised TAMs and the inhibition of TAM-associated molecules [26, 27].

Along with TAMs, MDSCs are characterised by the capacity to suppress T cell functions and support tumour progression [28]. MDSCs are a heterogenous group of immune cells from the myeloid lineage, particularly enriched in the peripheral blood, bone marrow (BM), and neoplastic tissues in cancer patients, exerting a suppressive effect on antitumour immunity, by inhibiting both innate and adaptive immune reactions [29]. These cells comprise at least two subsets: monocytic MDSCs (identified as CD11b+Ly6G−Ly6Chi cells in mouse and CD11b+CD14+HLA−DRlow/−CD15− cells in human) and granulocytic MDSCs (PMN-MDSCs, identified as CD11b+Ly6G+Ly6Clo cells in mouse and CD11b+CD14−CD15+ or CD11b+CD14−CD66+ cells in human) [28]. Accumulation of myeloid progenitors and their differentiation to TAMs and MDSCs is the result of a process driven by cancer-related inflammation, involving: altered myelopoiesis; mobilisation of myeloid precursors from the BM; recruitment of MDSCs and TAMs precursors into both secondary lymphoid organs and/or tumour tissues; functional diversion of myeloid cells in response to microenvironmental signals. This multistep process drives the reprogramming of myeloid cells towards a *tumour*-promoting phenotype and remotely controls the composition of the tumour-microenvironment. Previous studies demonstrated that a decrease in immunosuppressive MDSCs leads to the activation of host immunity, and that several chemotherapy drugs, including fluorouracil and gemcitabine, reduced the numbers of MDSCs in tumour-bearing hosts [30]. In addition, immunostimulatory molecules, including CpG oligodeoxynucleotide and polyinosinic-polycytidylic acid (polyI:C), which are ligands of toll-like receptors (TLRs) and/or retinoic acid–inducible gene-I (RIG-I)–like receptors, inhibit the immunosuppressive functions of MDSCs and/or induce differentiation of MDSCs into immune-activating cells, including macrophages [31]. MDSC’s function has been studied in breast cancer. These studies demonstrated that MDSCs induce inhibition of T cells, NK cells and DCs, while stimulating immune regulators such as Th2 T cells, T-reg, and TAMs. MDSCs also secrete cytokines such as IL-6 and IL-4 allowing MDSC expansion and subsequent sequestration of essential amino acids such as arginine and cysteine, which are required for the survival of T cells. MDSCs are also thought to mediate their inhibitory functions through reactive oxygen species such as nitric oxide [29].

Recently, the involvement of extracellular vesicles (EVs) in cancer pathophysiology has been described and, given their release from all cell types under specific stimuli, have also been proposed as potential biomarkers in cancer. In particular, the role of exosomal-PD-L1 fraction has been recently hypothesiSed as a possible determinant of response to ICI, in patients with lung cancer and melanoma [32]. Exosomes are nano-sized membrane vesicles produced in the endosomal cell compartment and, together with microvesicles (MVs) generated by budding from the plasma membrane and apoptotic bodies, represent EVs circulating component [33]. The role of exosomes as local and systemic cell-to-cell possible mediators of oncogenic information, through horizontal transfer of various bioactive molecules, such as proteins and mRNAs, has been recently hypothesised in BC [34]. Our pilot preliminary data confirmed previously reported data [35], demonstrating that the overall blood concentration of EVs resulted significantly higher in cancer patients than in healthy volunteers. Moreover, as expected, we detected PD-L1+ EVs only in the tumour, lymphoid and endothelial compartment of breast cancer patients. Preclinical studies also support the role of exosomes in TME and host immunity, and suggest exosomes as potential multi-functional therapeutic agents for TNBC [33]. Moreover, Piao *et al* [36], using an orthotopic TNBC model, found that TNBC-derived exosomes were able to polarize macrophages, favouring lymph node metastasis, by modifying tumour microenvironment. Recently, it has been shown that exosomes have the potential to change the fate of macrophage phenotypes, either M1, classically activated macrophages, or M2, alternatively activated macrophages on the basis of their molecular cargo [37, 38]. In terms of therapeutics, exosomes cargoes can be artificially modulated to provide pro-inflammatory, anti-tumourigenic microenvironment to suppress the tumour growth and metastasis.

The reactivation of immunosurveillance is critical for better prognosis and improved patient survival; to this purpose, it has been developed a strategy to induce ICD, a type of tumour cell death, which primes an anticancer immune response. In response to ICD, tumour cells expose calreticulin (CRT) on the cell surface prior to death and release DAMP molecules, such as adenosine triphosphate (ATP) during apoptosis or HMGB1 upon secondary necrosis, which stimulate the recruitment of DCs into the tumour bed, the uptake and processing of tumour antigens, and the antigen presentation to T cells [39]. As a consequence, the increase in the number of TILs, with high ratio CD8+/Treg, and activated CD8+, may elicit a direct cytotoxic response to eradicate cancer cells, through the generation of IFNg, perforin-1, and granzyme B [40] (Figure 1).

A lot of chemotherapeutic drugs have been used to induce ICD [39], but in the recent years, the interest has focused on oncolytic virotherapy as a strategy to stimulate ICD-hallmarks production, particularly IFN, which takes advantage of competent replicating viruses to destroy cancer cells.

## Oncolytic virotherapy

**Figure 2.**
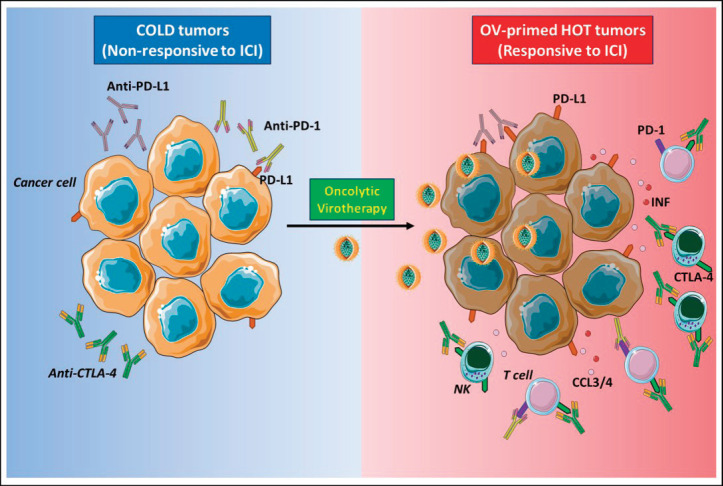
Oncolytic viruses make tumours ‘hot’. ‘Cold’ tumours poorly infiltrated by immune cells and have low expression of PD-L1 on cancer cells surface, and for this reason, the response to ICI therapy is inefficient (left panel). Oncolytic virotherapy promotes strong antiviral immune response accompanied by the production of cytokines, such as IFN type I that stimulates PD-L1 expression on the surface of cancer cells, and chemokines, like CCL3 and CCL4, attract immune cells expressing PD-1 or CTLA-4. These events make tumour ‘hot’, and when ICIs are administered subsequently, they can bind to their respective targets on either cancer or immune cells (right panel). NK: Natural killer; PD-1: Programmed death-1; PD-L1: Programmed death ligand-1; CTLA-4: Cytotoxic T-lymphocyte-associated protein 4; IFN I: Interferon type I; CCL3/4: Chemokine (C-C motif) ligand 3 or 4. The figure was modified from Servier Medical Art, http://smart.servier.com/.

The aim of oncolytic virotherapy is to stimulate anti-cancer immune response, targeting and killing tumour cells while sparing normal cells. In this perspective, natural or engineered viruses, able to transform a ‘cold’ into a ‘hot’ TME, with increased immune cell and cytokine infiltration (Figure 2). Yet, several clinical trials are evaluating the combination of oncolytic virotherapy and ICI [41]. In the clinical setting, intravenous oncolytic Orthoreovirus has been shown to increase T cell infiltration in brain tumours and to up-regulate IFN-regulated gene expression and PD-L1 expression, creating a favourable TME for subsequent ICI therapy [42]. In melanoma patients, the sequential administration of an attenuated herpes simplex virus type 1 (HVS-1), and an anti-PD-1 therapy resulted in a 33% complete response rate, with increased CD8+ T cells, PDL1 protein and IFNg gene expression in responders. Baseline CD8+ T cell infiltration or a baseline IFNg signature was not associated with response [43]. Oncolytic viral therapy is therefore a potential future option to skew the TME towards an ICI responsive phenotype.

### Oncolytic viruses (OVs)

OVs are RNA- or DNA-attenuated viruses able to kill tumour cells after having infected them, inducing TME remodelling and antitumour immune response. Most available oncolytic viruses are genetically modified to reduce virulence for non-neoplastic host cells.

Since 1904 many researchers have been studying the relationship between viruses and tumour cells [44, 45], focused on virus growth in tumours, rather than studying the effects of these viruses on tumour growth. The first clinical trial was reported in 1940, when an attenuated virus against melanoma led to a remarkable partial remission [46], and in 1949, the first preclinical study described the ability of the Russian Far East Virus to inhibit the growth of five transplanted mouse sarcoma 180 [47]. In 1965, Lindenmann and Klein [48] demonstrated that enhanced humoral immunity against tumour cell antigens, via the secretion of immunoglobulin and cytotoxic antibodies, was due to post-oncolytic immunity. In the next years, several viruses were studied in various leukaemia models, Hodgkins’ disease and Burkitt’s lymphoma associated with measles infection.

With the first experiences with recombinant DNA technology, in 1991, the first demonstration that herpes simplex virus (HSV) mutants (dlsptk), with depleted thymidine kinase or Infected cell protein (ICP) 34.5, were selectively replicating only in proliferating cells of human glioma xenograft was achieved [49].

To date, nine different families of viruses, including both DNA and RNA viruses, have successfully transitioned from preclinical studies to early clinical trials, with more than 570 ongoing clinical trials testing OVs [10]. This activity has now granted the approval of Talimogene Laherparepvec (T-VEC), a modified oncolytic HSV-1 for clinical use in US, Europe and Australia [50], along with the clinical use of adenovirus-derived Oncorine for head and neck cancers treatment in China [51] and native Echovirus 7 (Rigvir) for the treatment of melanoma in several European countries [52].

### Mechanisms of oncolytic virus immunotherapy

OVs mediate toxic effect on tumour cells by direct or indirect mechanisms. This activity is influenced by the efficiency of cell receptor targeting, viral replication and host cell antiviral response. The lytic potential of oncolytic viruses also depends on the type of virus, dose, natural and induced viral tropism, and the susceptibility of the cancer cell to the different forms of cell death (apoptosis, necrosis, pyroptosis and autophagy) [7].

**Figure 3:**
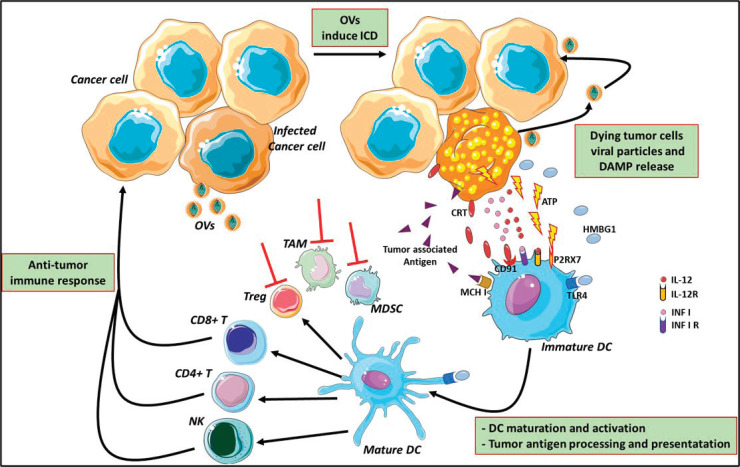
Mechanism of oncolytic virus immunotherapy. Oncolytic viruses infect cancer cells and induce the immunogenic cell death and release of infectious viral progeny that infect nearby cancer cells. Tumour-associated antigens and cellular DAMPs, such as CRT, HMGB1 and cellular ATP, stimulate the host antitumour immune responses. Cellular detection of viral infection and the products of oncolysis trigger the rapid activation of host antiviral responses and influx of immune cells that mediate the destruction of residual infected and uninfected tumour cells. The direct recognition and killing of tumour cells is primarily mediated by natural killer cells of the innate immune system and tumour antigen-specific CD8+ cytotoxic and CD4+ helper T lymphocytes of the adaptive immune system. The effects of OVs reflect on MDSCs, TAMs and Treg cells inhibition and modify the suppressive microenvironment altering the cytokine milieu (red lines) OVs: oncolytic viruses; ATP: adenosine triphosphate; HMGB1: high mobility Group Box 1; CRT: calreticulin; DAMPs: damage-associated molecular pathways; CD: cluster differentiation; TLR4: Toll-like receptor 4; P2RX7: Purin 2 Receptor X7; DC: dendritic cell; MCH I: major histocompatibility complex Class I; Treg: T regulatory; TAMs: tumour-associated microenvironment; MDSCs: myeloid-derived suppressor cells. The figure was modified from Servier Medical Art, http://smart.servier.com/.

In normal cells, pathogen-associated molecular patterns (PAMPs), such as elements of viral capsids, DNA, RNA and viral protein products, active intracellular TLRs pathway, coordinate with the antiviral machinery in infected cells; this reinforces local IFN release, which, in turn, activates Protein Kinase R (PKR). PKR is a protein kinase, encoded by the EIF2AK2 gene, activated by double-stranded RNA (dsRNA) and introduced to the cells by a viral infection. It can induce cell death and viral clearance [53]. In cancer cells, IFN pathway signalling and PKR activity may be abnormal; thus, viral clearance is prevented, allowing increased viral replication. Moreover, the inherent abnormalities in the cancer cell response to stress, cell signalling and homeostasis provide a selective advantage for viral replication and in cancer cells, excluding normal cells [53]. As a consequence of oncolytic cell death, cancer cells also release viral PAMPS and additional ICD-hallmarks (DAMPs; for example, heat shock proteins, HMGB1, CRT, ATP and uric acid) and cytokines (e.g., type I IFNs, tumour necrosis factor-α (TNFα), IFNγ and interleukin‑12), which promote the maturation of antigen-presenting cells (APCs) such as DCs (Figure 3). DCs are considered a ‘bridge’ between innate and adaptive immunity and, for this reason, may serve as a carrier for some OVs (such as reovirus and measles) protecting from neutralising antibodies [54]. DCs have an important role in the initiation of immune responses, to control and eliminate viral infections. Yet, they activate the innate immune cells (such as NK) to respond to pathogens through non-specific mechanisms [55]. In particular, ‘immature’ DCs upon pathogen recognition or stimulation become ‘mature’, expressing co-stimulatory molecules (CD40 and B7), homing receptors (i.e. CCR7) and producing cytokines which induce their migration from peripheral blood to secondary lymphoid organs, where they process antigen and exhibit it *via* MHC class I-II, promote the expression of corresponding costimulatory molecules on T cells (CD40L, CD28) [56] and, finally, prime T cells activation and differentiation. There are different DC subsets distinguished from CD expression (CD8, CD4, CD103 and CD11b). Different DC subsets induce different T helper (Th) cell polarization [57].

Cancer cells can overcome the immune-mediated attack by modifying TME, facilitating the recruitment of immune-suppressive cells, such as TAMs and MDSCs. OVs can modify this suppressive microenvironment through a variety of mechanisms that alter the cytokine milieu and the type of immune cells within the TME potentiating systemic immunity against the cancer [58]. Moreover, oncolytic cell death can induce the release of novel cancer antigens (neo-antigens) that may have been previously hidden from the immune system, an effect already known with ICI [59, 60]. The presence of these neo-antigens is important since it may trigger an immune response and the newly generated T cell clones may be able to circulate and kill antigen-expressing cancer cells, including cancer cells that were not infected by the virus. The nearby uninfected tumour cells may be killed by the cytotoxic perforins and granzymes released by the infected tumour cells [61].

### Oncolytic virus transport and biodistribution

One of the major issues to use the OVs in therapy is the route of administration and needs to take into account physical barriers that can reduce the spread of OVs, such as the presence of necrosis, calcification, hypoxia, acidosis, increased proteolytic activity, high interstitial pressure, blood–brain barrier, tumour size and heterogeneity, dense extracellular matrix and poorly vascularisation [62]. Intratumoural (IT) injections is the main strategy used in the majority of clinical studies with OVs since it allows maximal delivery of high viral titres to tumours, bypassing systemic neutralisation and premature clearance. The main disadvantage is represented by physical inaccessibility for those tumours that are not accessible by clinical palpation; in this case, localisation via interventional imaging or surgical exposure might be required, which may be challenging for OVs repeated administrations over time. The ability of OVs to induce systemic anti-tumour response, as discussed in the previous section, could be a solution to overcome this limitation, as evidenced in the T‑VEC OPTIM phase III clinical trial [9.] In the case of Seneca Valley Virus (SVV) [63] or the chimeric adenovirus, enadenotucirev [64], delivery by intravenously (IV) administration has been shown to allow viral distribution at any site, precluding the need for additional training and interventional procedures associated with IT delivery. In the case of SVV, it can be delivered IV because of natural resistance to hemagglutination, a process resulting in premature viral clearance and reduced delivery to the tumour site following intravenous delivery [63]. The issues with IV delivery of any OVs includes considerable dilution in the systemic circulation and the likelihood of premature neutralisation through serum anti-viral immunoglobulins or other serum proteins, especially after multiple infusions. Of note, viruses naturally cause viremia and are more susceptible to antibodies neutralisation; thus, the intravenous administration of these viruses may limit the effects in patients who have had previous treatments or vaccination. For this reason, it is important to assess the viral presence not only in the tumour lesion but also in the blood. Normally, tumour specimens are tested for the presence of virus using IHC and confirmed by PCR assay. However, PCR is a highly sensitive assay for viral sequences, but have not a confirmation of live viral particles and on-going replication and more informative test are been required. In recent years, the sensitivity and accuracy of real-time PCR assays have been greatly improved by the droplet digital PCR technology (ddPCR), whose detection limit is significantly lower when compared to that of real time PCR [65].

Different approaches, such as the use of cell carriers, immunomodulators and liposomes have also been proposed. Yet, dendritic and T cells [54], mesenchymal stem cells [66] or carcinoma cells [67], could be a carrier of viruses to tumour sites, also in the presence of neutralising antibodies. The concomitant administration of immunomodulatory drugs, such as cyclophosphamide, seems to rescue OVs [68]. Clinical trials are ongoing to understand if OVs could be considered in combination or as a neoadjuvant therapy with cyclophosphamide [8]. Liposomes were also used in pre-clinical studies to encapsulate oncolytic alphavirus strain M1 (M-LPO) [69] and ONYX-015-based plasmid [70]. Liposomes harbouring the plasmids were resistant to antibodies neutralising the parent strain, while the plasmids could only transfect tumour cells which are p53 deficient [70].

Recent studies highlight the importance of MVs in cancer development. These specialised MVs are able to transfer bioactive molecules from one cell to another [33]. Tang *et al* [71] have demonstrated that tumour cell-derived MVs (T-MV), containing both tumour antigens and innate signals, can serve as safe and efficient carriers to deliver chemotherapeutic drugs to tumour cells. Moreover, they reported that T-Mvs can be utilised as a unique carrier system to deliver oncolytic adenoviruses to human tumours, leading to highly efficient cytolysis of tumour cells needed for *in vivo* treatment efficacy [72]. Exosomes are MVs (100–150 nm) that can influence viral infection in different ways, in relation to type of virus and cells. For example, exosomes isolated from human immunodeficiency virus (HIV) infected cells contained negative regulatory factors, transactivation response elements, viral microRNAs (vmiR-88, vmiR-99 and vmiR-TAR), CCR5, and CXCR4—all of which facilitate HIV-1 invasion [73]. Hepatitis C virus (HCV) utilises exosomes to transfer its genomic RNA, antisense RNA, and proteins, which aid the virus in establishing a productive infection [74]. Exosomes can also confer membranes to non-enveloped viruses, such as hepatitis A virus, allowing to escape host immune recognition. Exosomes deriving from liver nonparenchymal cells can transmit interferon (IFN)-α induced antiviral activity to hepatitis B virus replication hepatocytes [75].

### Development of oncolytic viruses as drugs

The rules of a well-designed OV approach are mainly two: 1) to attenuate the viral pathogenesis and 2) to target and kill tumour cells, selectively. Natural tropism for surface molecule, altered cancer cell pathways together with the flexibility of recombinant engineering have allowed the investigation of several strategies to enhance the effectiveness of OVs. There are some molecules that are overexpressed on cancer cell surface (CD46, CD155, CD54, CD55, alfa2beta1, laminin receptor, etc.) and that can be used by OVs to recognise and entry into target cells. Some OVs, like HSV-1 and Coxsackievirus, naturally recognise the overexpressed molecules (herpesvirus entry mediator – HVEM- and CD54, respectively) and can enter in the cancer cell [76, 77]; other OVs can be engineered to directly target unique cell surface receptors, as in the case of adenovirus Ad5/3‑Δ24, which was modified to bind to integrins that are highly expressed on ovarian cancer cells [78].

A long intracellular incubation time is needed to allow OV replication and spread, explaining the preference for tumour cells infection, characterised by aberrant signalling pathways that promote resistance to cell death and proliferation. For example, Newcastle disease virus (NDV) targets B cell lymphoma (BCL) [79] or Reovirus and Vaccinia virus show natural selectivity for cells with overexpression of the RAS signalling pathway [80]. Furthermore, the possibility to insert viral genes under active promoters in cancer cells, allows limiting viral replication only in cancer cells. This is the case of a phase I trial for patients with prostate cancer, treated with adenovirus (CV706) in which the E1A, an adenoviral protein that inhibits cell cycle, was placed under promoter of prostate-specific antigen (PSA) [81]. In prostate cancer cells the promoter of PSA is highly active, E1A is selectively expressed only in these cells, resulting in proliferation of adenovirus and virus mediated cell lysis [81].

Since normal and cancer cells exhibit differential expressions of cognate miRNA elements, the engineered OVs with miRNA Target Sequencing (miRTA) can be blocked from replicating in normal cells, where specific miRNAs are expressed. This type of construct was designed in glioma, where it showed limited viral replication in neurons that constitutively express high levels of miR‑7, while allowing proliferation in glioma cells, that frequently downregulate miR‑7 [82]. Recent evidence has demonstrated that exosomal miRNA can not only be secreted by cancer cells but it can also be uptake by these cells. It has been also hypothesised that NDV might use exosomes to entry into neighbouring miRNA carrying cells, resulting in inhibition of the IFN pathway and promotion of viral infection [83].

## Oncolytic virus therapy in breast cancer

In recent years, the role of ICI has been extensively studied in breast cancer patients, with particular emphasis on triple negative disease leading to FDA and EMA approval of atezolizumab in the first line treatment of PD-L1 overexpressing TN MBC [5]. Moreover, extensive research is ongoing to characterise the prognostic role of TILs [84]. In this perspective, a powerful strategy to enhance the response to ICI, such as anti-PD1/PD-L1, is TME reprogramming: yet, the administration of chemotherapy agents, such as doxorubicin and cisplatin, has been shown to induce up-regulation of inflammatory JAK-STAT and TNF-α signalling and to increase responsiveness to Nivolumab in metastatic TNBC [85]. Innovative treatment options, such as OVs, are studied as a monotherapy or in combination with ICI or CT, both in pre-clinical and clinical trials.

### Oncolytic Herpes Simplex Virus (HSV)

HSV‑1 is a double-stranded DNA virus with a large genome (152 kb), member of the alphaherpesvirus family; it replicates in the nucleus and is not able to induce insertional mutagenesis, making HSV‑1 an attractive candidate for therapeutic development. Human breast cancer cells are permissive to HSV-1 and genetic manipulations allow virus selectivity, while sparing normal cells [86].

Talimogene laherparepvec (T‑VEC) is the most commercially advanced HSV‑1‑ based OV. T‑VEC contains deletions of two genes: ICP34.5 and ICP47. ICP34.5 is a neurovirulence gene, critical for blocking the host antiviral PKR–IFN response; its deletion confers cancer selectivity and prevention of neural infection. The two ICP34.5 genes have been substituted with the gene encoding granulocyte-macrophage colony-stimulating factor (GM-CSF) to improve the induction of antitumour immunity. ICP47 deletion results into viral antigen presentation, leading to the containment of the infection in healthy tissues and immune-mediated kill of cancer cells that selectively propagate oncolytic HSV‑1. The deletion of ICP47 also induces the early activation of the US11 promoter that blocks PKR phosphorylation preventing cancer cell apoptosis [86].

In preclinical studies, T‑VEC demonstrated potent lytic effects against several tumour cell lines, most notably melanoma and pancreatic cancer cells [10]. T‑VEC has been evaluated in clinical trials in patients with melanoma, pancreatic cancer, head and neck tumours, and in patients with breast cancer in a phase I clinical trial [87]. Treatment was generally well tolerated, with most adverse events related to fever, chills, nausea, fatigue and local injection site reactions.

T-VEC may represent a valuable tool in combination with ICIs, particularly in those tumours with a low baseline lymphocyte infiltrate that are poorly responsive to ICI as a single agent, such as breast cancer. A phase 1b clinical trial, including 21 advanced melanoma patients treated with T-VEC followed by pembrolizumab, showed an interesting level of activity of the anti-PD-1 agent, related to TME modulation [43]. The safety and efficacy of T-VEC (as a monotherapy or combination therapy with paclitaxel) in TNBC patients is under evaluation in a clinical trial [17]. Recently, antitumour activity of G47Δ-mIL12 was observed in a 4T1 tumour model of advanced breast cancer. G47Δ-mIL12 (14) is a genetically engineered oncolytic HSV (oHSV) that has similar genetic modifications to T-VEC but contains ICP6 inactivation, limiting HSV replication to cancer cells and expressing murine Interleukin 12 (IL-12) (instead of GM-CSF). The G47Δ-mIL12 efficacy has been tested both *in vitro* and *in vivo* experiments. *In vitro, it was able to* infect and eliminate both murine and human TNBC cells. *In vivo*, G47Δ-mIL12 treatment effectively inhibited 4T1 tumour growth in a CD8+ T cell-dependent fashion, and prevented metastatic spread [88].

Administration of HF10 in immunocompetent mouse models of colon and breast cancer stimulated specific antitumour immune responses and provided resistance to malignant cell re-implants [89]. HF10 is a spontaneously mutated HSV-1 and is expected to be a promising agent in combination with bevacizumab, since bevacizumab enhances viral distribution, increases tumour hypoxia and expands the population of apoptotic cells, therefore producing a synergistic antitumour effect [90]. An investigator-initiated phase I trial of HF10 enrolled six patients with cutaneous and subcutaneous metastatic breast cancer. A histological examination revealed fibrosis and tumour cell death with an infiltration of CD8+ and CD4+ T-cells around tumour islets, supporting the induction of an immune response [91].

### Oncolytic adenovirus

Adenoviruses are medium-sized (70–90 nm), non-enveloped icosahedral viruses with double-stranded DNA. Many types of immunologically distinct adenoviruses can cause infections in humans. Adenovirus expresses coxsackie-adenovirus receptor (CAR) and adenoviral early genes (encoded by E1A and E1B) that allow the cell infection targeting suppressors oncogene p53 and retinoblastoma-associated protein (pRb). Normal cells are protected by p53 and pRb activities, whereas in cancer cells these genes are compromised, and adenovirus can interfere with cell cycle proteins. Some of the strategies for modification of adenovirus are described, as attenuation, and the use of oncolytic adenovirus in clinical practice has registered very few adverse events.

Oncolytic adenovirus provides a novel therapeutic modality for breast cancer with its appropriate targeting property. Adenoviruses ONYX‑015, which has a deletion in the portion of E1B that inactivates p53, are currently investigated in a phase I trial in combination with etanercept (a recombinant dimer of the human TNF); so far, this study enrolled two patients with metastatic breast cancer, as well as other patients with different types of cancer [92]. Recent findings demonstrated that a novel recombinant adenovirus, targeting E2F-1 and IL-15, has an inhibitive effect on breast cancer proliferation [93]. The transcriptional factor E2F-1 plays a significant role in the control of cell cycle, proliferation and carcinogenesis, and it is higher expressed in breast cancer tissues compared with normal tissues, suggesting that E2F-1 may be an effective target for treatment. Also, by carrying human Interleukin-15 (IL-15) gene, a crucial cytokine enhancing the activity of macrophages and neutrophils, it may induce the production and activation of effector T cells and regulate the survival and proliferation of memory T cells, giving to the oncolytic adenovirus an immunomodulatory effect [93]. Other pre-clinical studies highlighted the importance of oncolytic adenoviruses engineered interfering lncRNA [94] or targeting TGF-β in cancer cells [95].

### Pelareorep (Reolysin®)

Reolysin (Oncolytics Biotech Inc., Calgary, Alberta, Canada) is a purified live replication-competent form of the reovirus serotype 3 Dearing strain. REOvirus (Respiratory Enteric Orphan), belonging to the genus Orthoreovirus, family Reoviridae, is a 70 nm, naturally occurring, ubiquitous, non-enveloped, an icosahedral shaped virus with a genome of 10 segments of double-stranded RNA. The application of reovirus as an oncolytic virus exploits the RAS pathway that is linked to the activation of PKR [96]. A phase II, randomised, clinical trial of pelareorep in combination with paclitaxel in patients with metastatic breast cancer has shown that this combination is more effective than paclitaxel alone [97]. Recently, in a pre-clinical study, it has been demonstrated that reovirus was able to induce an immune response against breast cancer cells when combined with anti-PD-1 therapy [98].

### Vaccinia virus

Vaccinia virus is a dsDNA virus and is a member of the poxvirus family; they can infect a wide range of cells entering by endocytic mechanism and replicating in the cytosol. For its large capacity of transgenes, easy manipulation, a good safety profile, and the inability to integrate into the host genome, the vaccinia virus is considered a good OV. Specifically, viral thymidine kinase, vaccinia growth factor and vaccinia type I IFN-binding protein (B18R) have been modified to increase cancer cell selectivity and lysis [99]. Based on the importance of the immune response and the ability of the vaccinia viral genome to accept large transgenes (25 kb), vaccinia virus has been engineered to express tumour antigens, T cell co‑stimulatory molecules and inflammatory cytokines [7]. Recently, an engineered oncolytic vaccinia virus ((VV)-iPDL1/GM) that co-expresses a PD-L1 inhibitor and GMCSF has been developed. This OV can secrete the PD-L1 inhibitor, that systemically binds and inhibits PD-L1 on tumour cells and immune cells but can activate tumour neoantigen-specific T cell responses, via GM-CFS stimulation. This provides a tumour-specific oncolytic immunotherapy for cancer patients, especially those resistant to PD-1/PD-L1 blockade therapy [100].

Moreover, the Western Reserve strain of vaccinia virus (VV), the most virulent strain of VV in animal models, has been engineered for tumour selectivity through two targeted gene deletions (vvDD). vvDD were administered *via* IT dose escalation in 16 patients with advanced solid tumours (first-in-human phase 1). The study report that it was well-tolerated, showing a selective antitumour activity [101].

### Newcastle disease virus

NDV is a single-stranded RNA enveloped avian paramyxovirus that ranges in size from 100 to 500 nm. NDV infects cells through plasma membrane fusion or through direct endocytosis of the virus. Cancer cell specificity is conferred by its sensitivity to type I IFNs and overexpression of BCL-XL. NDV induces apoptosis and directly activates the innate immune system through increased cytokine production (type I IFN, RANTES, IL‑12, and GM‑CSF) and improved antigen presentation. The NDV protein haemagglutinin-neuraminidase can act as a potent antigen that augments cytolytic T cell responses against infected cells. NDV-induced apoptosis results in the conversion of an immune-suppressive tumour microenvironment into a pro-inflammatory environ­ment that supports antitumour immune responses.

NDV infection usually has minimal clinical symptoms, making this virus optimal for cancer treatment. This is due to the fact that cancer cells are relatively permissive to NDV infection. I*n vitro* and *in vivo data have shown* the effectiveness of different strains of oncolytic NDV as novel strategy for breast cancer therapy. A newly developed recombinant NDV that expresses IL12 (rAF-IL12) has been observed to inhibit tumour growth significantly (52%) in treated mice [102]. Recently, it has been demonstrated that combining NDV virotherapy with glucose analogue 2-deoxyglucose (2-DG), a enhances the anti-tumour effect in human- and mouse-breast cancer cells [103]. These results open the possibility to apply the use of OVs and in particular NDV in different strategies to treat breast cancer. The encapsulation of NDV in exosomes has been proposed as an innovative approach for virus delivery. Several studies have shown that exosomes are involved in viral infections and in intercellular communication, through miRNA. In a recent study performed in HeLa cell line, it has been speculated that NDV used exosomes to entry into neighbouring cells, the so-called ‘recipient cell’, carrying miRNAs produced by the original cell, called the ‘parent cell’, after virus infection; this results in IFN pathway inhibition and promotion of viral infection of the ‘recipient cell’ [104].

Although numerous preclinical studies have suggested that NDV has antitumour activity against a wide variety of cancers, there are only a limited number trials in progress at present [105]. The effect of NDV (PV701) has been evaluated in two BC patients enrolled into a phase I trial. PV701 was IV injected twice at lower dosages followed by dose escalation. The treatment regimen was well tolerated [106].

### Other OVs

There are other viruses under investigation for their oncolytic activity both as a single agent as well as in combination with other anticancer agent: coxsackievirus, poliovirus, vesicular stomatitis virus (VSV), measles virus and Maraba virus.

In particular, coxsackievirus strains and poliovirus reported promising results, both *in vivo* and *in vitro,* with favourable toxicity profile [107–109].

Oncolytic measles viruses (oMV) derived from the attenuated Edmonston-B vaccine strain can recognise target cells through overexpression of specific molecules such as CD46, signalling lymphocytic activation molecule, the poliovirus receptor-related 4 protein and Nectin Cell Adhesion Molecule 4 (Nectin 4). Nectin 4 levels can be downregulated by specific miRNA (i.e., miR-31 and miR-128) with the impact on oMV infectivity *in vitro* and in xenograft models [110]. It has also been observed that the Aurora A kinase inhibitor alisertib enhanced oMV oncolysis *in vitro* and improved outcome *in vivo* against breast cancer xenografts [111]. Intriguingly, a group of researchers demonstrated that, using dendritic cells as a carrier for oMV, it is possible to overcome the problem of antibody neutralisation with promising results in terms of virus delivery to breast cancer cells [112]. To date there is one phase I clinical trial that is currently open (NCT01846091), whose primary aim is to evaluate the toxicity of this approach in MBC.

Oncolytic rhabdoviruses like Maraba and VSV have been shown to enhance NK cell–mediated killing of tumour cells, dendritic cell maturation, up-regulation of antigen presentation by tumour cells and production of proinflammatory cytokines and chemokines [113]. Maraba rhabdovirus has been tested in a variety of mouse tumour models [113] and is now being evaluated in phase 1/2 studies (NCT02285816 and NCT02879760).

## Conclusion

Viruses used as a weapon against cancer is one the revolutionary discoveries of recent years.

The two main advantages in the use of oncolytic virotherapy are: 1) its specificity for tumour cells and 2) its active role in triggering anti-tumour immunity already present, as in the case of ‘hot’ tumours, and that of systemic immune cells attracted in TME, in ‘cold’ tumours. However, if, on the one hand, this form of immunomodulation is characterised by moderate toxicity, increased specificity, and hopefully activity, some issues must be optimised in the development of OVs as a new class of drugs. In fact, appropriate dosages and schedules, pharmacodynamic assays, delivery systems and type of clinical trial designs need to be carefully assessed, especially in combination regimens with CT or ICIs, to improve performance and results. Furthermore, intermediate endpoints, such as predictive biomarkers. Patient selection must also be carefully evaluated in terms of immune system performance.

Oncolytic virotherapy is located in the context of personalised medicine, since its best application is associated to the interplay between human immune system and TME. Many clinical trials including oncolytic virotherapy are phase II/III trials (Table 1), due to the need to increase the knowledge on specific mechanisms between OVs, human immune system and tumour, mainly to prevent adverse events. Severe adverse events caused by oncolytic virotherapy have rarely been reported, while moderate adverse events can be controlled or disappear spontaneously, avoiding viremia. Today, the OVs are administrated *via* IT and IV delivery, although the most appropriate route of administration has not yet been defined, to reduce adverse events and enhance OVs efficacy. In clinical trials, IV delivery may be preferable to IT injection: this approach, in fact, simplifies the interventional procedures and improves systemic delivery to distant metastasis. However, IV administration is associated with excessive dilution and premature neutralisation through serum anti-viral immunoglobulins or other serum proteins. To overcome this, it is recommended to use rapid infusion schedules, with repeated high doses to avoid serum neutralising antibodies, and preferentially in combination with other anticancer therapies.

The development of new oncolytic virus for the treatment of breast cancer includes new viral vectors targeting tumour cells with high affinity without perturbating normal cells, improving immune system activity against cancer, reducing adverse events and ensuring safety. In particular, in breast cancer, novel reovirus, vaccinia virus and HSV are been developing. Among these, vaccinia virus is considered to be very promising candidate, due to its transfection capacity, easy manipulation, good safety profile and inability to integrate into the host genome.

At present, there are several phase I and II clinical trials using OVs to treat breast cancer patients (Table 1); most of these use a combination approach. On the other hand, a number of preclinical trials are studying alternative combination approaches concerning delivery systems and dosages. In conclusion, oncolytic virotherapy seems to show good promise for breast cancer therapy, with potentially effective and well-tolerated regimens, and possibly improving quality of life.

## Conflicts of interest

There are no conflicts of interest.

## Figures and Tables

**Table 1:** Clinical Trials for breast cancer treatment using oncolytic virotherapy.

ID	Ststus	Phase	Actual enrolment	Type of disease	Virus	Additional Theraphy
NCT00574977	completed	I	26	BC, Melanoma, HNSCC, LC, CRC, PanA	vvDD-CDSR (VV)	none
NCT00636558	completed	I	8	BC, Melanoma, PC	CVA21 (Coxsackievirus)	none
NCT01017185	completed	I	28	BC, refractory HNSCC, Skin, melanoma	HF10 (HSV)	none
NCT01656538	completed	II	81	MBC	Reolysin (Reovirus)	Paclitaxel
NCT01846091	active, not recruiting	I	12	ER-, ER+, HNSCC, HER2/Neu-, HER2/Neu+, MBC, PR+, PR-, Stage IV BC, TNBC	MV-NIS (MV)	none
NCT02285816	recruiting	I/II	56	MBC, NSCLC, Esophageal and Gastric Cancer	MG1MA3 (oncolytic Maraba) and AdMA3 (AD vaccine)	none
NCT02630368	recruiting	I/II	118	MBC, Advance Tissue Sarcoma	Jx-594 (oVV)	metronomic cyclophosphamide
NCT02779855	active, not recruiting	I/II	50	Ductal BC, Ductal Carcinoma	Talimogene Laherparepvec (oHSV)	Paclitaxel (neoadjuvant)
NCT02977156	recruiting	I	66	TNBC, Mesothelioma, HNSCC, CRC	Pexa-Vec (oVV)	lpilimumab
NCT03004183	recruiting	II	57	TNBC, NSCLC	ADV/HSV-tk (oAd)	Pembrolizumab, stereotactic XRT
NCT03564782	recruiting	I	6	Stage II-IV TNBC	PVSRIPO (oncolytic poliovirus)	none

**Abbreviations:** Ad: Adenovirus; HSV: Herpes Simplex virus; MV: Measles Virus; VV: Vaccinia Virus; MV-NIS (MV): Oncolytic Measles Virus Encoding Thyroidal Sodium Iodide Symporter; TNBC: Triple Negative Breast Cancer; MBC: Metastatic Breast Cancer; HNSCC: Head neck Squamous Cell Carcinoma; LC: Liver Carcinoma; CRC: Colorectal Cancer; PanA: Pancreatic Cancer; PC: Prostate Cancer; ER: Estrogen Receptor; PR: Progesterone Receptor; NSCLC: Non Small Cell Lung Cancer.
